# Whole-Genome Sequence of *Streptococcus tigurinus* Strain osk_001, Isolated from Postmortem Material

**DOI:** 10.1128/genomeA.00878-17

**Published:** 2017-08-31

**Authors:** Hidenori Yoshizawa, Daisuke Motooka, Ryuichi Katada, Yuki Matsumoto, Shota Nakamura, Eiichi Morii, Tetsuya Iida, Hiroshi Matsumoto

**Affiliations:** a Department of Legal Medicine, Osaka University Graduate School of Medicine, Osaka, Japan; b Project for Death Control and Prevention, Osaka University Graduate School of Medicine, Osaka, Japan; c Osaka Prefectural Medical Examiner’s Office, Osaka, Japan; d Department of Pathology, Osaka University Graduate School of Medicine, Osaka, Japan; e Department of Infection Metagenomics, Research Institute for Microbial Diseases, Osaka University, Osaka, Japan

## Abstract

*Streptococcus tigurinus* was recently described as a novel species, and some strains are highly virulent. We detected *S. tigurinus* in infected tissue sampled by necropsy. In order to characterize and confirm the virulence of this species, whole-genome sequencing of the pure cultured bacterium was performed. We found that the strain has specific and unique genetic elements contained in highly virulent strains of *S. tigurinus*.

## GENOME ANNOUNCEMENT

*Streptococcus tigurinus* was recently described as a novel species, originally identified by Zbinden et al. Some strains of *S. tigurinus* are highly virulent and cause serious invasive infections, with or without bacteremia, such as infective endocarditis, meningitis, spondylodiscitis, and necrotizing fasciitis (1–3). Diene et al. reported that specific and unique genetic elements are contained in highly virulent strains of *S. tigurinus* (4). We recently diagnosed a severe soft-tissue infection at the Osaka Prefectural Medical Examiner’s Office and detected *S. tigurinus* in a necropsy sample. The isolate was cultured and designated *S. tigurinus* strain osk_001. When performing postmortem microbiology during an autopsy or necropsy, microorganisms isolated from a cadaver’s tissue should be carefully analyzed to determine whether they are truly pathogenic and causative of the diagnosed infection (5). Therefore, we performed whole-genome sequencing of the *S. tigurinus* strain osk_001 to characterize and confirm the virulence in this case.

Complete genome sequencing of *S. tigurinus* strain osk_001 was performed using a combination of the MiSeq (Illumina) and PacBio RS II (Pacific Biosciences) platforms. Genomic DNA of *S. tigurinus* was extracted from cultured cells using a PowerSoil DNA isolation kit (Mo Bio). For MiSeq sequencing, 500 ng of genomic DNA was sheared to about 600 bp, the library was prepared using KAPA library preparation kits (KAPA Biosystems), and then paired-end sequencing (251 bp × 2) was performed. For PacBio RS II sequencing, 2 μg of genomic DNA was sheared to about 15 kb, the library was prepared using a DNA template prep kit (version 1.0), and sequencing was performed. PacBio reads were *de novo* assembled to a contig using HGAP (6). In order to correct sequence errors, MiSeq reads were mapped onto the assembled PacBio contig using CLC Genomics Workbench version 9.5.3 (CLC bio/Qiagen). This contig was trimmed and circularized into the final complete genome of *S. tigurinus* osk_001, which comprised 1,889,005 bp with a G+C content of 41.15%. A total of 1,831 predicted coding DNA sequences, 12 rRNAs, and 59 tRNAs were annotated with MiGAP.

Whole-genome sequencing revealed that *S. tigurinus* strain osk_001 included some of the same genetic elements present in highly virulent strains. We concluded that *S. tigurinus* strain osk_001 is not a low-virulent strain and that this bacterium may be pathogenic and causative of severe soft-tissue infections. Whole-genome sequencing appears to be a useful tool for confirming whether the microorganisms detected by postmortem microbiology are truly pathogenic and causative of diagnosed infections.

### Accession number(s).

The genome sequence for *S. tigurinus* strain osk_001 has been deposited at DDBJ/GenBank under accession no. AP018338.
